# *Lactiplantibacillus plantarum-encapsulated* microcapsules prepared from okra polysaccharides improved intestinal microbiota in Alzheimer’s disease mice

**DOI:** 10.3389/fmicb.2024.1305617

**Published:** 2024-03-18

**Authors:** Yao-Kun Hsiao, Bao-Hong Lee, She-Ching Wu

**Affiliations:** ^1^King Long Guan Company Ltd., Chiayi, Taiwan; ^2^Department of Food Sciences, National Chiayi University, Chiayi, Taiwan; ^3^Department of Horticultural Science, National Chiayi University, Chiayi, Taiwan

**Keywords:** okra polysaccharides, microcapsules, microbiota, short-chain fatty acids, Alzheimer’s disease

## Abstract

**Background:**

Okra contains a viscous substance rich in water-soluble material, including fibers, pectin, proteoglycans, gum, and polysaccharides. This study explored the use of okra polysaccharides by microorganisms and their potential to improve microbiota.

**Methods:**

The regulation of microcapsules prepared from okra polysaccharides with or without *L. plantarum* encapsulation on intestinal microbiota was assessed through 16S metagenomic analysis and short-chain fatty acids (SCFAs) in App*^NL-G-F/NL-G-F^* mice (Alzheimer’s disease; AD model).

**Results:**

We found that *Lactobacillaceae* and *Lactobacillus* were majorly regulated by microcapsules prepared from okra polysaccharides in AD mice. Similarly, microcapsules prepared from okra polysaccharides with *L. plantarum* encapsulation markedly elevated the abundance of *Lactobacillaceae* and *Lactobacillus* and increased SCFAs in AD mice.

**Conclusion:**

Our results suggest that microcapsules prepared from okra polysaccharides with or without *L. plantarum* encapsulation may improve intestinal microbiota by elevating *Lactobacillus* levels in AD mice.

## 1 Introduction

There are approximately 10^13^–10^14^ microbial cells in the human gastrointestinal tract, which is 10 times the total number of human cells; the total number of genes expressed by microorganisms exceeds the total number of human genes expressed by approximately 150 times or more (Qin et al., 2020). In terms of taxonomy, 50 phyla of microorganisms have been discovered and defined, of which 10 are found in the gut, including primarily *Bacteroides* spp., followed by *Actinobacteria* and *Proteobacteria* (Rajilic-Stojanovic et al., 2011).

The goblet cells and enterocytes in the gut produce mucin and mucus, and many gut microbes can use these substances as a source of growth nutrients (prebiotics) for their own growth and reproduction and to produce short-chain fatty acids (e.g., acetic acid, propionic acid, butyric acid, and valeric acid). Microorganisms that have this ability (e.g., *Akkermansia muciniphila* and *Faecalibacterium prausnitzii*) are known as next-generation probiotics, and their role is valued due to the ability of short-chain fatty acids to inhibit intestinal pathogens (Machiels et al., 2014; De la Cuesta-Zuluaga et al., 2017).

In addition to being consumed as a vegetable, okra (*Abelmoschus esculentus*) is also used in traditional medicine (Lengsfeld et al., 2004; Sengkhamparn et al., 2009; Sami et al., 2013). Okra contains a viscous substance that is rich in polysaccharides. Studies have reported that okra contains water-soluble fibers, such as pectin, proteoglycans, and gum arabic, which can be used as thickeners in cooking and food preparation (Woolfe et al., 1977; BeMiller et al., 1993), and have the characteristics of high viscosity, strong stability, and good suspension (Romanchik-Cerpovicz et al., 2002). Chemical analysis revealed that okra polysaccharides are composed of rhamnose, xylose, galactose, and arabinose (Sami et al., 2013). An early study extracted viscous substances from okra via acid hydrolysis and found that galactose, rhamnose, and galacturonic acid are the major carbohydrates in okra (Whistler and Conrad, 1954). It has also been suggested that the viscous substances extracted from okra are composed of acidic polysaccharides, proteins, and minerals. After hydrolysis, the ratios of galacturonic acid, galactose, rhamnose, and glucose obtained by colloidal chromatography were 1.3:1.0:0.1:0.1, respectively (Woolfe et al., 1977). In recent years, many research reports have pointed out that okra polysaccharides have neuroprotective functions, which can reduce nerve damage caused by amyloid beta and metabolic syndrome (Huang et al., 2020; Yan et al., 2020b; Huang et al., 2023). Due to the increasing attention to the relationship between neurodegenerative diseases and gut microbiota (Zhou et al., 2012), although there are relevant literature reports on the effect of okra polysaccharides on neurological diseases, the potential of okra polysaccharides to regulate gut microbiota to alleviate neurodegenerative diseases still needs further evaluation. Therefore, this study utilized App*^NL-G-F/NL-G-F^* mice to investigate the ability of okra polysaccharides to modulate the gut microbiota composition.

To date, large numbers of studies have explored the relationship between gut microbiota and Alzheimer’s disease (AD), and many intestinal microbes have been found to accelerate its progression (Zhou et al., 2012). These microbes are often associated with metabolic diseases; a high proportion of Firmicutes/Bacteroidetes microbes is linked to intestinal inflammation and an increase in the incidence of AD (Soscia et al., 2010; Cowan et al., 2014; Chen et al., 2016). In the App*^NL-G-F/NL-G-F^* mouse model, in which Aβ accumulates without the overexpression of APP, leading to artificial phenotypes, Aβ deposition starts at 2 months of age, and plaques accumulate over time (Sakakibara et al., 2018). There are three mutations within the Aβ sequence in APP peptides, Aβ amyloidosis is aggressively accelerated, and neuroinflammation is observed in subcortical structures and cortical regions. These observations consistently indicate that App*^NL-G-F/NL-G-F^* mice represent a model for preclinical AD and are useful for studying AD prevention rather than treatment after neurodegeneration (Sakakibara et al., 2018). Several studies have explored the relationship between intestinal flora and AD. Many intestinal microorganisms have been found to accelerate AD progression, and these microorganisms are usually associated with metabolic diseases. Chen et al. (2016) confirmed that the amyloid protein produced by microorganisms in the intestine enters the central nervous system through the bloodstream and increases the aggregation and deposition of Aβ.

Because okra polysaccharides are mucin-like polysaccharides, these macromolecules are similar to mucin and mucus secreted by human intestinal cells (enterocytes and goblet cells) and may maintain the intestinal barrier and prevent the penetration of microorganisms into the intestinal lumen. When harmful bacteria exist in large quantities in the intestinal tract, the epithelial cells of the intestinal tract lose their normal physiological functions, resulting in inflammation and increased permeability of intestinal cells (gut permeability), a phenomenon known as leaky gut (Turner, 2009). The viscous substance in okra contains polysaccharides, which can be used as a material to promote the growth of probiotics to improve intestinal health. Okra polysaccharides have the ability to modulate the gut microbiome and thus are able to inhibit inflammation (Yan et al., 2020a,b). Microcapsules are materials with encapsulating structures that can protect their contents. In many healthy products, microcapsules are used to encapsulate probiotics to increase their survival ability in the intestine. Carbohydrates and polysaccharides are commonly used materials for preparing microcapsules. In this study, okra polysaccharides were used as a common material for encapsulating probiotic (*Lactiplantibacillus plantarum*) microcapsules. Additionally, the intestinal protective effect of okra polysaccharides against gut microbiota dysfunction was evaluated in this study.

## 2 Methods

### 2.1 The preparation of okra polysaccharides

The dried experimental okra stored at 4°C was taken out and pulverized, and the extraction was carried out with hot water for 3 h with double distilled water. After extraction, sedimentation was carried out with 4-fold the volume of 95% ethanol, and finally, the sediment was dried in a freeze dryer; the obtained powder is okra crude polysaccharide, which is stored in a 4°C refrigerator for the determination of polysaccharide content and subsequent experiments. To ensure whether the crude okra polysaccharide has residual protein, take 50 μL of okra polysaccharide aqueous solution, mix it with 200 μL of protein quantitative analysis solution (Bio-Rad, United States) in a 96-well culture plate, and observe its color change for 2 min. The original solution of protein quantitative analysis solution is reddish-brown, and the Coomassie Brilliant Blue G-250 contained in it will combine with protein and turn into blue, which can confirm no protein contamination in crude okra polysaccharide.

### 2.2 Assay for polysaccharides properties

Polysaccharide concentrations were determined using the phenol–sulfuric acid method (Dubois et al., 1956). Glucose solutions (0, 25, 50, 75, 100, and 125 mg/L) were prepared for use as standards. Then, 1 mL of glucose standard solutions (different concentrations) or sample (okra polysaccharides) were mixed with 0.5 mL of 5% phenol and 2.5 mL of sulfuric acid and allowed the reaction for 20 min at room temperature. The absorbance was measured at 490 nm with spectrophotometer, and the concentration of polysaccharides was calculated from the standard curve. The molecular weight of okra polysaccharides was identified by nuclear magnetic resonance (NMR). The okra polysaccharides were fluorescently labeled according to the manual (SugarLighter, New Taipei City, Taiwan). In brief, adlay polysaccharide powder (1 mg) was mixed with hydrolysis solution (1 mL) for reaction at 80°C for 2 h. The solution was mixed with 2 mg 2,3-naphthalenediamine, 1 mg iodine, and 1 mL acetic acid (stirring for 1 h) for monosaccharide fluorescent labeling (Lin et al., 2010). The molecule weight was measured by diffusion ordered NMR spectroscopy (DOSY, Bruker AV600) at 600 MHz to obtain the diffusion coefficient and H1 profile (Lee et al., 2023).

### 2.3 *Lactiplantibacillus plantarum* culture

*Lactiplantibacillus plantarum* were purchased from the Bioresource Collection and Research Center (BCRC11697) in Taiwan (Hsinchu, Taiwan). These lactic acid bacteria were inoculated into MRS broth (BD Biosciences, San Jose, Calif., United States) or MRS agar at 37°C and transferred monthly. This strain has been reported to exhibit anti-cancer (Liu and Pan, 2010) and use in fermentation (Kuo et al., 2021).

### 2.4 Preparation of okra polysaccharide microencapsules

The okra polysaccharides were mixed with sodium alginate (4.5, 5, and 5.5%) in different proportions to prepare wall material solution. Subsequently, the core material solution (2 x 10^10^CFU/mL lactic acid bacteria suspension) was mixed with wall material solution at 1: 1 (w/w). This mixed solution was transported and separated by gas and solidify by 5% CaCl_2_ for 20 min for microcapsulation (Nasiri et al., 2021). The crystal microcapsules were washed and frozen-dried.

### 2.5 Analysis of microencapsule properties

For encapsulation efficiency, the okra polysaccharides were used as the stock solution and diluted with deionized water into five different concentrations, and then, the absorbance was measured with a spectrophotometer to make a standard calibration. The absorbance of okra polysaccharide microcapsules was measured, and the encapsulation efficiency was calculated according to a study by Nasiri et al. (2021). For swelling capacity, the 0.3 g of sample into a 10 mL graduated cylinder, add 10 mL of distilled water and let stand at room temperature for 24 h, then read the volume of the impregnated sample (Ralet and Della, 1993). For the mechanical strength test, the microencapsules (3 g) were stained by crystal violet and add 30 mL of deionized water. This microcapsules solution was stirred at 400 ± 10 rpm for 180 min, and the morphology and broken were observed and calculated.

### 2.6 Animals treatment

The App*^NL-G-F/NL-G-F^* mice contained a humanized amyloid beta (Aβ) region and introduced the Swedish mutation and the Beyreuther/Iberian mutation into the mouse amyloid precursor protein (APP) gene. This mouse model will overexpression Aβ42 and a high Aβ42/40 ratio, which causes cognitive decline at 6 months. Animals (10 months old) were divided into three groups (*n* = 6/group), including Group-A: transgenic (TG) mice group (App*^NL-G-F/NL-G-F^* mice), Group-B: TG mice administered with microcapsules (200 mg/kg bw) prepared from okra polysaccharides were mixed with sodium alginate (5%), and Group-C: TG mice administered with *L. plantarum*-encapsulated microcapsules (200 mg/kg bw) prepared from okra polysaccharides were mixed with sodium alginate (5%). The dosage (200 mg/kg bw) of okra polysaccharides was accorded to a study, which could show bioactivity (Liao et al., 2019). All animals were kept in the cages at 25 ± 2°C under the 12-h/12-h light/dark cycle, and the relative humidity was 55 ± 10% with free to water and fed. Experimental animals used in this study had been reviewed and approved by the Institutional Animal Care and Use Committee (IACUC) in National Cheng Kung University in Taiwan with IACUC approval No. 108317. At the end of the experiment, the mice were deprived of food for 12 h and were euthanized with carbon dioxide. Intestinal feces were collected and stored at −20°C until analyzed.

### 2.7 Assays for intestinal microbiota

Feces in the colon of each mouse (n = 6) were collected and immediately soaked in liquid nitrogen and stored at −80°C for subsequent use. The total genomic DNA from samples was extracted using QIAamp PowerFecal DNA Kit (Qiagen) for 16S gene sequencing. V3–V4 region was amplified by specific primer set (319F, 5’-CCTACGGGNGGCWGCAG-3′, 806R, 5’-GACTACHVGGGTATCTAATCC-3′) according to Illumina MiSeq PE300 platform. In brief, 12.5 ng of gDNA was used for the PCR reaction under the conditions: 95°C for 3 min; 25 cycles of: 95°C for 30 s, 55°C for 30 s, 72°C for 30 s; 72°C for 5 min and hold at 4°C. 16S analysis using QIIME2 alignment platform^1^ with SILVA132 annotation database.

### 2.8 Assay for short-chain fatty acids

The levels of SCFAs such as acetic acid, propionic acid, and butyric acid were performed by gas chromatography–flame ionization detection (GC-FID) that used the Shimadzu GC-2010 (Shimadzu Corp, Tokyo, Japan) with a capillary column (BP21 FFAP 30 m × 0.53 mm i.d., 0.50 μm film thickness, Trajan, Melbourne, Australia). The carrier gas was nitrogen, and the split less injection volume was 1 μL. Auxiliary gasses for the flame ionization detector were hydrogen (30 mL/min of flow rate) and dry air (300 mL/min of flow rate). The temperatures of the injector and detector were 220°C and 240°C, respectively. The temperature of the GC oven was first set at 90°C for 1 min and elevated to 150°C at 10°C/min, then to 200°C at 20°C/min, and following held for 1 min. Triplicate repeats were analyzed, and the obtained data were normalized to the concentrations of external standards and are shown in μM (Lee et al., 2021a).

### 2.9 Statistical analysis

The experiments were performed in five repeats, and the results are expressed as the mean ± standard error of mean. The results were examined by using one-way analysis of variance and Duncan’s multiple range tests, and the significance of differences between sample means was calculated. A *p*-value of ≤0.05 was considered significant. For β diversity of microbiota analysis, pairwise analysis of similarities (ANOSIM) with permutations were conducted and evaluated using principal coordinate analysis (PCoA) based on different distance matrices, where *p*-values were reported after Benjamini–Hochberg multiple testing correction. The value corresponding to the heatmap represents the Z-score obtained by the abundance of each species in all groups. The Z-score of a sample on a certain classification is the value of the average abundance of the sample on the category and all samples in the classification. Linear discriminant analysis (LDA) effect size (LEfSe) uses non-parametric factorial Kruskal–Wallis (KW) sum-rank test to find species with significant differences in abundance and uses linear regression discriminant analysis (LDA) to estimate the impact of abundance of each species to find out the communities or species that have a significant difference in the sample division. LEfSe analysis of the screening value of LDA score is set to 4.

## 3 Results

### 3.1 Molecular weight of okra polysaccharides

**Figure 1:**
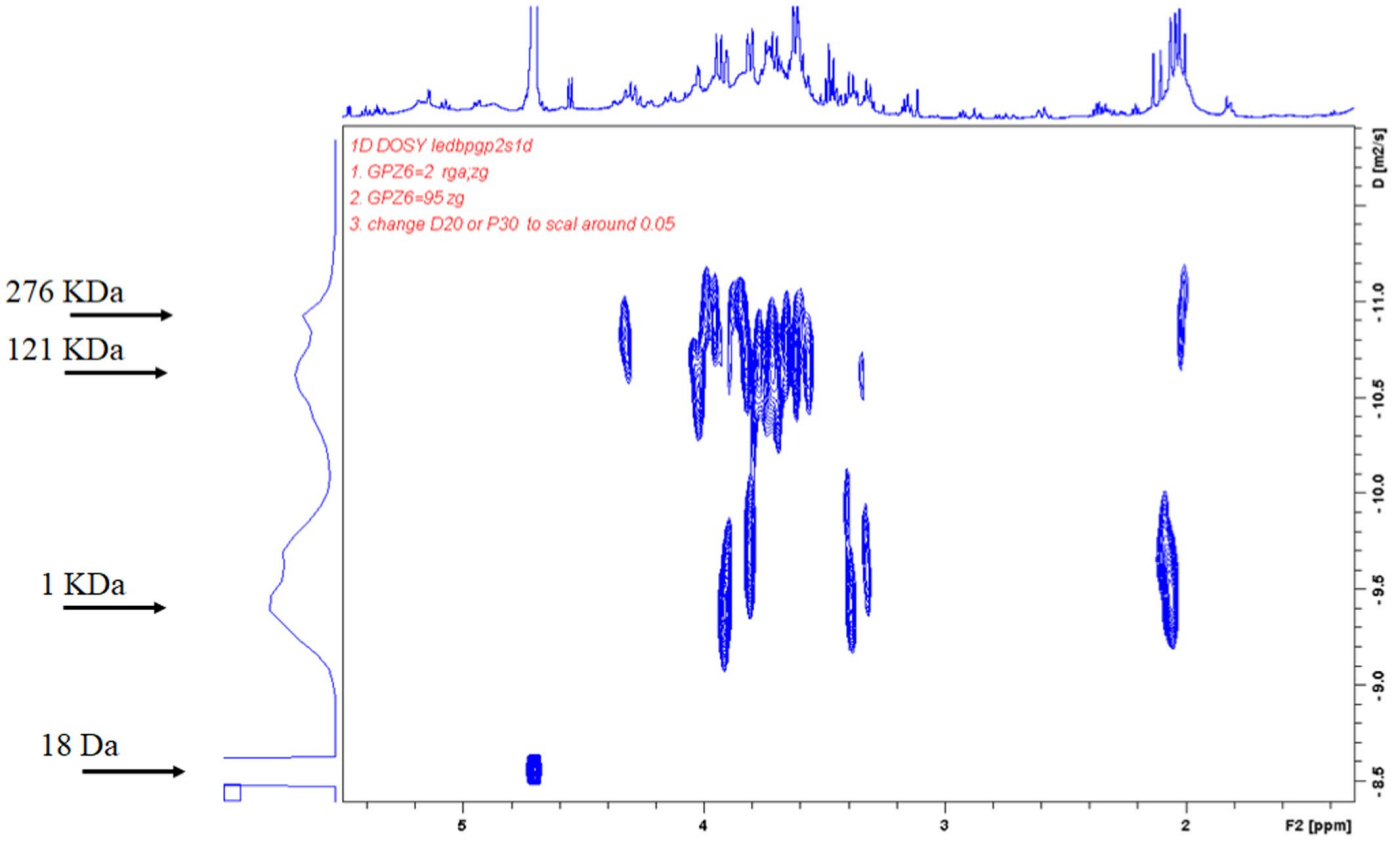
Diffusion-ordered NMR spectroscopy (DOSY) spectrum of adlay polysaccharides. Average molecular weight of purified adlay polysaccharides was determined and shown by 2D DOSY spectra. The horizontal axis represents the peak of NMR hydrogen ion absorption, and the vertical axis represents the self-diffusion coefficients. The molecular weight of polysaccharides ranges from 1 KDa to 2,767 KDa, and the highest peak of diffusion coefficient is the molecular weight of 1 KDa. The spectrum of 18 Da is solvent (H_2_O).

On analysis of the composition of okra polysaccharides, the proportion of water-soluble polysaccharides was found as 84% (W/W). The okra monosaccharide composition was composed of glucose (20.1%), mannose (16.7%), galactose (19.2%), arabinose (13.5%), xylose (5.7%), fructose (16.7%), and rhamnose (4.2%) (Table 1). The molecular weight distribution of okra polysaccharides by NMR spectrum has been performed. The molecular weight of polysaccharides ranges from 1 KDa to 2,767 KDa, and the highest peak of diffusion coefficient is the molecular weight of 1 KDa. The spectrum of 18 Da is solvent (H_2_O) (Figure 1).

**Table 1:** Monosaccharide composition.

Monosaccharide composition	Ratio (%)
Glucose	20.1 ± 1.5
Mannose	16.7 ± 2.1
Galactose	19.2 ± 1.4
Arabinose	13.5 ± 0.8
Xylose	5.7 ± 0.3
Fructose	16.7 ± 2.7
Rhamnose	4.2 ± 0.6

Data are shown as mean ± SD (*n* = 3).

### 3.2 Analysis of microcapsules properties

**Figure 2:**
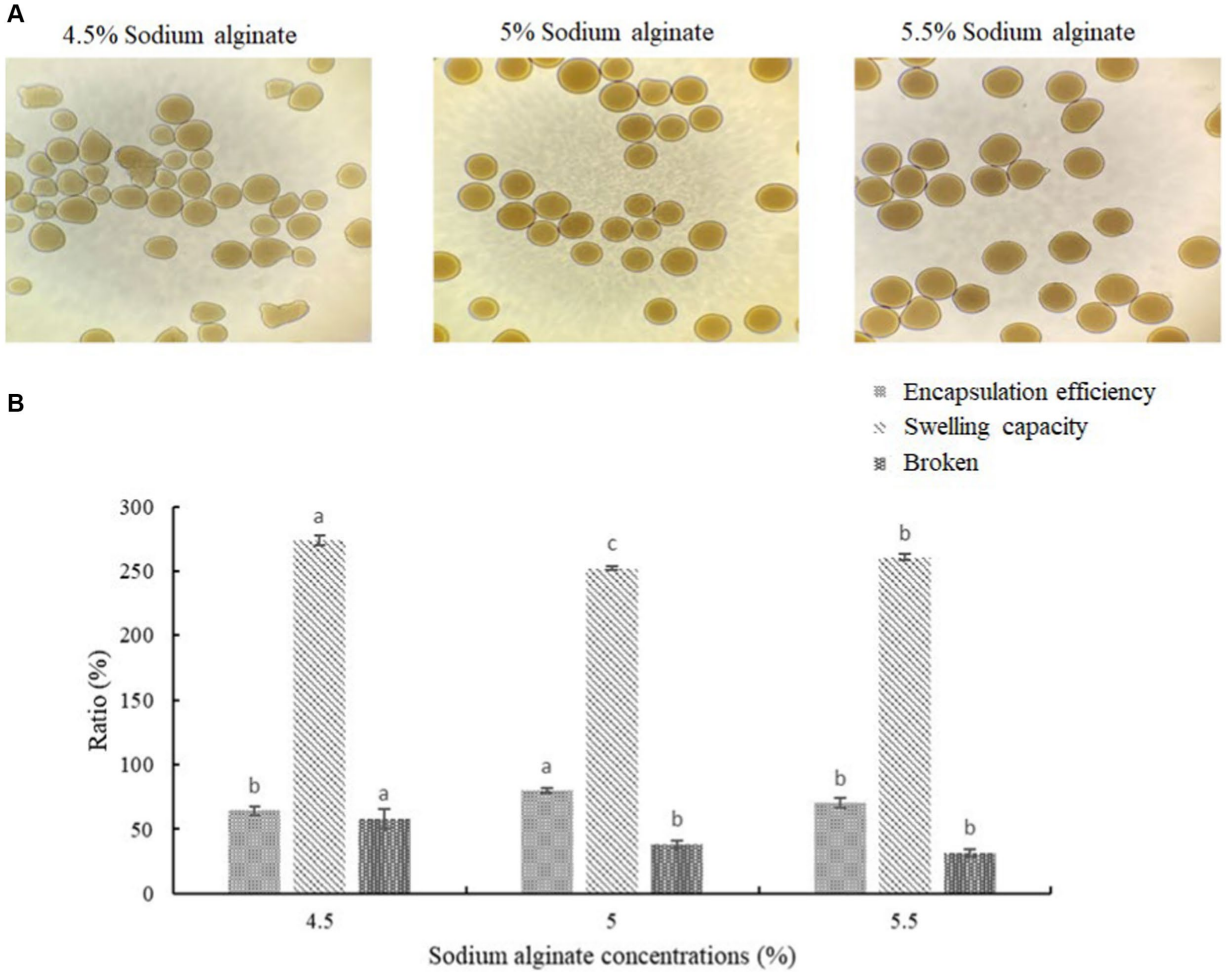
Effects of different sodium alginate concentrations on appearance of okra polysaccharide microcapsules. **(A)** Morphology of microcapsules prepared by 4.5, 5%, or 5.5% sodium alginate with okra polysaccharides. **(B)** Encapsulation efficiency, swelling capacity, and mechanical strength test (broken) of okra polysaccharide microcapsules with different sodium alginate concentrations. *Each value is expressed as mean ± S.D. (*n* = 3). Each value on the top right of means in the same column bearing of different letters is significantly different (*p* < 0.05).

The microcapsules were prepared from okra polysaccharides with different concentration of sodium alginate (4.5, 5, and 5.5%). We found that low concentration (4.5%) of sodium alginate resulted in serious tailing phenomenon in microcapsules, which may cause of low viscosity to lead to incomplete formation. From these results, it can be concluded that the mixed colloid with a sodium alginate concentration of 5.0% is the most suitable condition for the preparation of okra polysaccharide microcapsules (Figure 2A). The encapsulation efficiency, swelling capacity, and mechanical strength test of microcapsules prepared from okra polysaccharides with different concentration of sodium alginate were evaluated (Figure 2B). The encapsulation efficiency is related to the concentration of the wall material, the properties of the wall material, and the interaction between chemical molecules. The results showed that highest encapsulation efficiency (79.86%) could be found in microcapsules prepared from okra polysaccharides with 5% sodium alginate. The microcapsules were mixed with water, and then, the swelling capacity was investigated. The results for moisture content and particle size of microcapsules prepared from okra polysaccharides with sodium alginate are shown in Supplementary Table S1. The different particle sizes (214 μm, 237 μm, and 248 μm) were found in microcapsules by various concentrations of sodium alginate (4.5, 5, and 5.5%). We found that the concentration of sodium alginate markedly affected swelling capacity (4.5% > 5.5% > 5%) in microcapsules prepared from okra polysaccharides. In addition, the mechanical strength test was evaluated, which suggested that 5 and 5.5% sodium alginate could elevate resistance against microcapsules broken (Figure 2B).

### 3.3 Effects of administration with microcapsules prepared from okra polysaccharides with or without *Lactiplantibacillus plantarum* encapsulation on intestinal microbial diversity in App*^NL-G-F/NL-G-F^* mice

**Figure 3:**
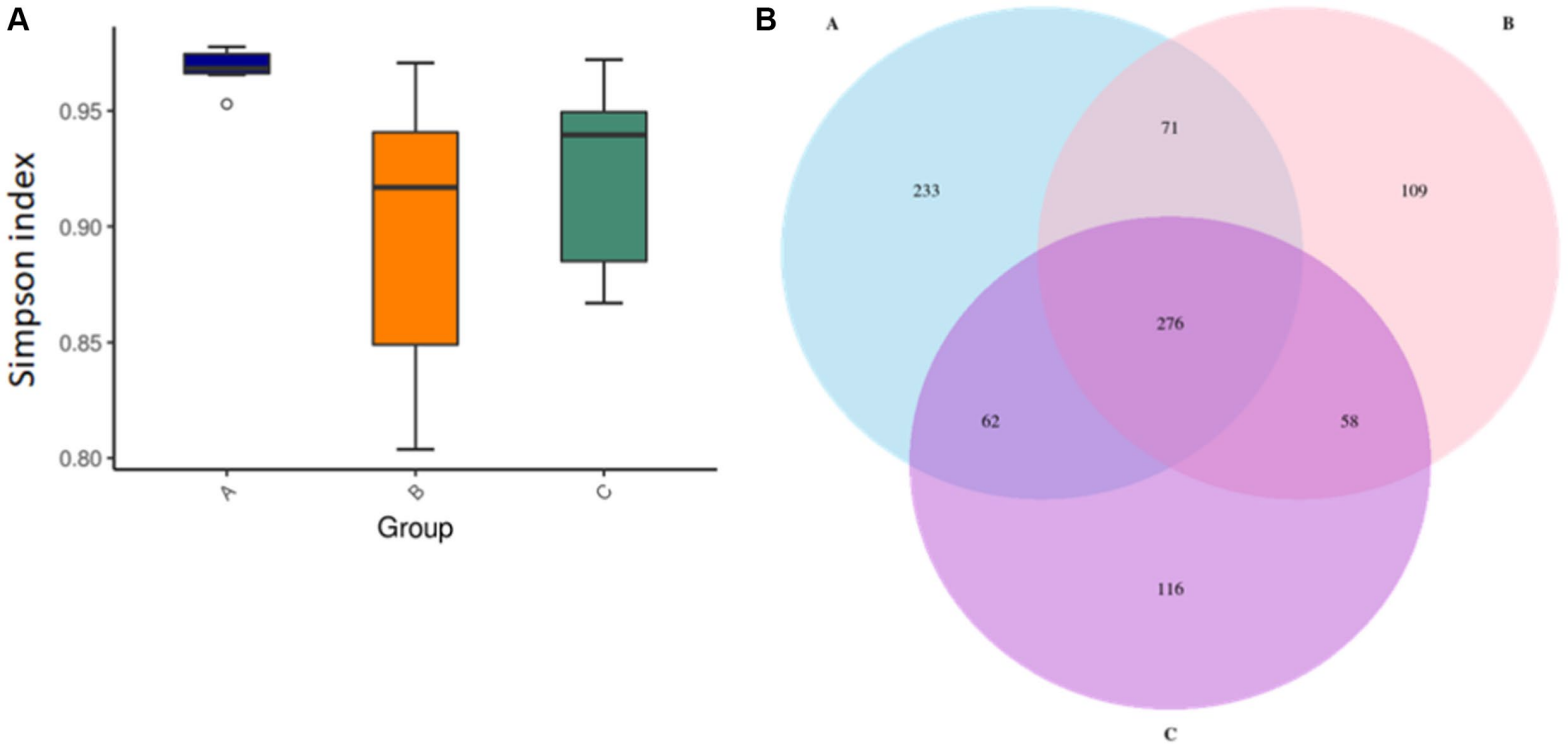
Assay for alpha diversity in intestinal microbiota in mice administered with microcapsules prepared from okra polysaccharides with or without *L. plantarum* encapsulation. **(A)** The Simpson indexes of alpha diversity obtained from the analysis of bacterial microbiota between groups. **(B)** Venn diagrams of analysis for the microbial OTU in each group. A: App*^NL-G-F/NL-G-F^* transgenic mice; B: App*^NL-G-F/NL-G-F^* transgenic mice administered with microcapsules (200 mg/kg bw) prepared from okra polysaccharides were mixed with sodium alginate (5%); C: App*^NL-G-F/NL-G-F^* transgenic mice administered with *L. plantarum*-encapsulated microcapsules (200 mg/kg bw) prepared from okra polysaccharides were mixed with sodium alginate (5%).

**Figure 4:**
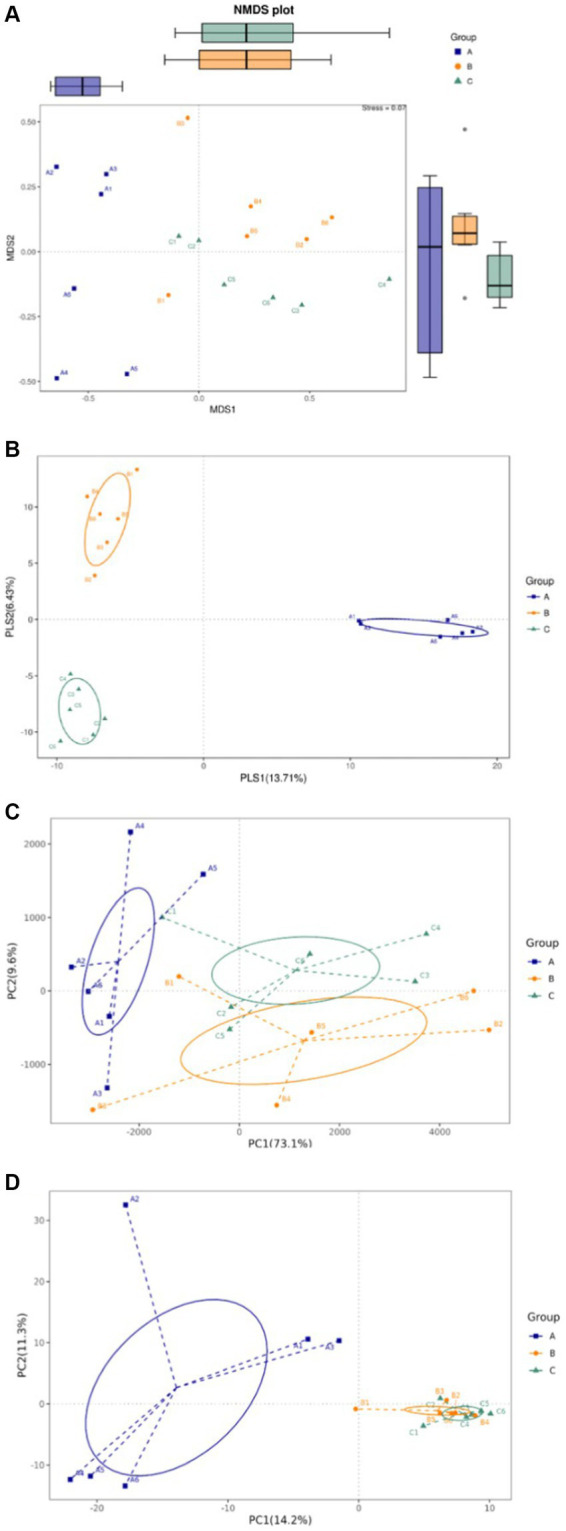
Assay for beta diversity in intestinal microbiota in mice administered with microcapsules prepared from okra polysaccharides with or without *L. plantarum* encapsulation. **(A)** Non-metric multidimensional scaling (NMDS), **(B)** partial least squares discriminant analysis (PLSDA), **(C)** principal component analysis (PCA)-covariance matrix, and **(D)** PCA correlation matrix. A: App*^NL-G-F/NL-G-F^* transgenic mice; B: App*^NL-G-F/NL-G-F^* transgenic mice administered with microcapsules (200 mg/kg bw) prepared from okra polysaccharides were mixed with sodium alginate (5%); C: App*^NL-G-F/NL-G-F^* transgenic mice administered with *L. plantarum*-encapsulated microcapsules (200 mg/kg bw) prepared from okra polysaccharides were mixed with sodium alginate (5%).

The intestinal flora is closely related to the health of the human body and the development of diseases. In healthy individuals, the microbiota in the intestinal environment originally has a balanced bacterial composition, with imbalance arising in conjunction with aging, stress, environment, and dietary habits. The composition of the intestinal flora changes and causes some flora to form dominant species in the intestinal ecological environment. To assess whether okra polysaccharides have potential to regulate intestinal microbiota *in vivo*, the fecal bacteria of mice were subjected to 16S metagenomic analysis in this study. The Simpson index was used to evaluate microbial alpha diversity in the intestine, and the index was negatively correlated with diversity. The AD mice were administered with microcapsules prepared from okra polysaccharides with or without *L. plantarum* encapsulation which increased bacterial alpha diversity compared to the AD mice (Figure 3A). Moreover, 233, 109, and 116 different bacteria (operational taxonomic units; OTU) were found in AD mice (Group-A), AD mice administered with microcapsules (200 mg/kg bw) (Group-B), and AD mice administered with *L. plantarum*-encapsulated microcapsules (200 mg/kg bw) (Group-C). However, there are 276 bacteria co-occurred among these three groups (Figure 3B). Beta diversity is a differential analysis of the microbial composition between different groups. According to the species annotation, the ASV information was merged for members belonging to the same classification group to obtain the species abundance. We used non-metric multidimensional scaling (NMDS), partial least squares discriminant analysis (PLSDA), and principal component analysis (PCA), including correlation matrix (PCA_corr) and covariance matrix (PCA_cov), to assess beta diversity. Our results indicate that both microcapsules prepared from okra polysaccharides (200 mg/kg bw) and *L. plantarum*-encapsulated microcapsules (200 mg/kg bw) could increase beta diversity in AD mice (Figure 4).

### 3.4 Effects of administration with microcapsules prepared from okra polysaccharides with or without *Lactiplantibacillus plantarum* encapsulation on intestinal microbiota in App*^NL-G-F/NL-G-F^* mice

**Figure 5:**
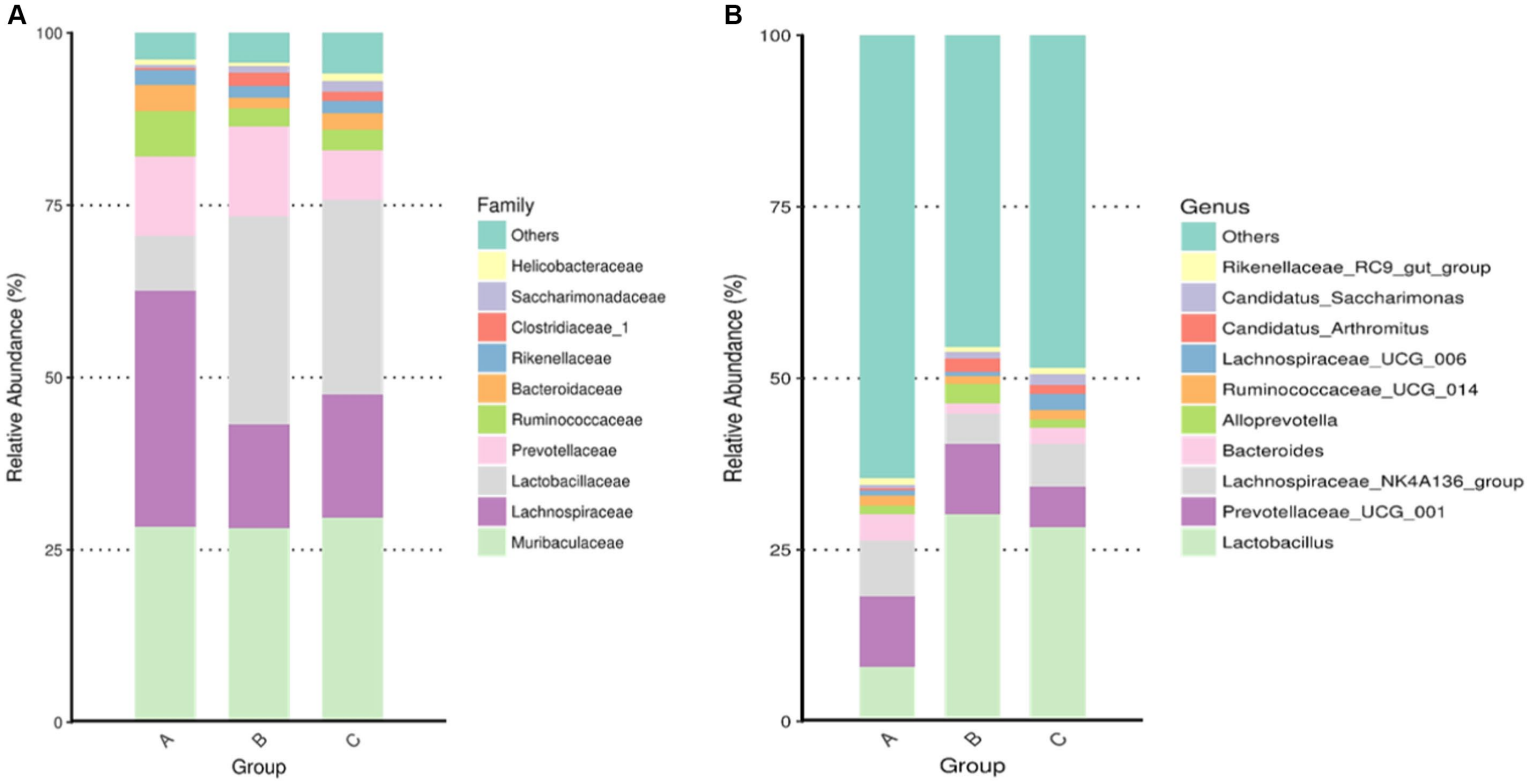
Assay for intestinal microbiota in mice administered with microcapsules prepared from okra polysaccharides with or without *L. plantarum* encapsulation. The top-10 abundance from taxa analysis of microbiota composition at **(A)** family and **(B)** genus. A: App*^NL-G-F/NL-G-F^* transgenic mice; B: App*^NL-G-F/NL-G-F^* transgenic mice administered with microcapsules (200 mg/kg bw) prepared from okra polysaccharides were mixed with sodium alginate (5%); C: App*^NL-G-F/NL-G-F^* transgenic mice administered with *L. plantarum*-encapsulated microcapsules (200 mg/kg bw) prepared from okra polysaccharides were mixed with sodium alginate (5%).

**Figure 6:**
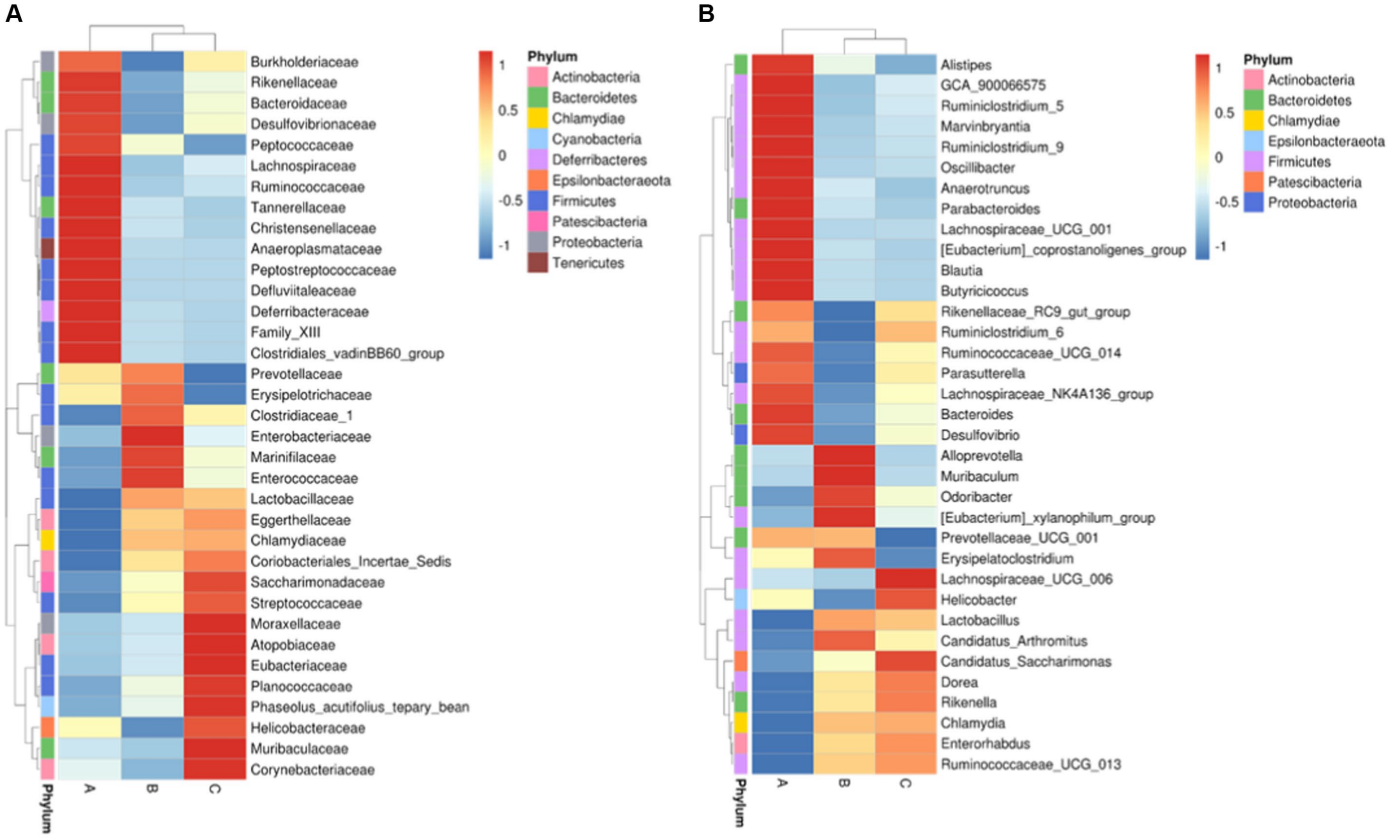
Intestinal taxa microbiota composition for **(A)** family level and **(B)** genus level in mice administered with microcapsules prepared from okra polysaccharides with or without *L. plantarum* encapsulation by heatmap. A: App*^NL-G-F/NL-G-F^* transgenic mice; B: App*^NL-G-F/NL-G-F^* transgenic mice administered with microcapsules (200 mg/kg bw) prepared from okra polysaccharides were mixed with sodium alginate (5%); C: App*^NL-G-F/NL-G-F^* transgenic mice administered with *L. plantarum*-encapsulated microcapsules (200 mg/kg bw) prepared from okra polysaccharides were mixed with sodium alginate (5%).

Among the top-10 most abundant microbial families and genera, our results indicated that the levels of *Lachnospiraceae*, *Ruminococcaceae*, and *Bacteroidaceae* in AD mice were higher than that in AD mice administered with microcapsules prepared from okra polysaccharides with or without *L. plantarum* encapsulation. However, the administration of microcapsules prepared from okra polysaccharides with or without *L. plantarum* encapsulation elevated *Lactobacillaceae* (family) and *Lactobacillus* (genus) levels in AD mice (Figure 5). We performed an analysis of intestinal microbiota at the genus and species level using a heatmap showing the ratio of intestinal microbiota in mice administered with microcapsules prepared from okra polysaccharides with or without *L. plantarum* encapsulation compared to the AD mice. Figure 6 shows the top 35 families and genera in each group included in the heatmap plot. The results indicated an increase in the intestinal abundance of *Burkholderiaceae*, *Rikenellaceae*, *Bacteroidaceae*, *Desulfovibrionaceae*, *Peptococcaceae*, *Lachnospiraceae*, *Ruminococcaceae*, *Tannerellaceae*, *Christensenellaceae*, *Anaeroplasmataceae*, *Peptostreptococcaceae*, *Defluvitaleaceae*, *Deferribacteraceae*, and *Clostridiales_vadin* BB60_group (family) in AD mice. We also observed a greater abundance of genera including *Alistipes*, *Ruminiclostridium*_5, *Marvinbryantia*, *Ruminiclostridium*_9, *Oscillibacter*, *Anaerotruncus*, *Parabacteroides*, *Lachnospiraceae*_UCG_001, *Blautia*, *Butyricicoccus*, *Rikenellaceae*_RC9_gut_group, *Ruminiclostridium*_6, *Ruminococcaceae*_UCG-014, *Parasutterella*, *Lachnospiraceae*_NK4A136_group, *Bacteroides*, and *Desulfovibria* in AD mice. However, these bacteria were suppressed in AD mice administered with microcapsules prepared from okra polysaccharides with or without *L. plantarum* encapsulation, even though populations of *Alloprevotella, Muribaculum, Odoribacter*, and *Lactobacillus* were increased in AD mice treated by microcapsules prepared from okra polysaccharides. We also found that the *Lachnospiraceae*_UCG_006, *Lactobacillus, Candidatus saccharimonas, Dorea, Rikenella,* and *Enterorhabdus* levels were increased in AD mice by administering microcapsules prepared from okra polysaccharides with *L. plantarum* encapsulation. *Lactobacillaceae* and *Lactobacillus* are mainly affected by microcapsules prepared from okra polysaccharides treating AD mice. Similarly, microcapsules prepared from okra polysaccharides with *L. plantarum* encapsulation markedly elevated the abundance of *Lactobacillaceae* and *Lactobacillus* in AD mice (Supplementary Figure S1). A study has also reported similar results, administration of lactic acid bacteria elevated the abundance of *Bifidobacterium*, *Bacteroides*, *Alistipes*, and *Alloprevotella*, but the numbers of *Parabacteroides, Lachnoclostridium, Lachnospiraceae* UCG-006, *Eubacterium,* and *Romboutsia* were reduced in high-fat diet-induced mice (Li et al., 2020). Our results suggested that microcapsules prepared from okra polysaccharides with or without *L. plantarum* encapsulation elevated the levels of lactic acid bacteria (*Lactobacillales, Lactobacillaceae,* and *Lactobacillus*) and maintain intestinal microbiota (*Atopobiaceae, Actinobacteria, Coriobacteriia*, and *Coriobacteriales*) in AD mice.

**Figure 7:**
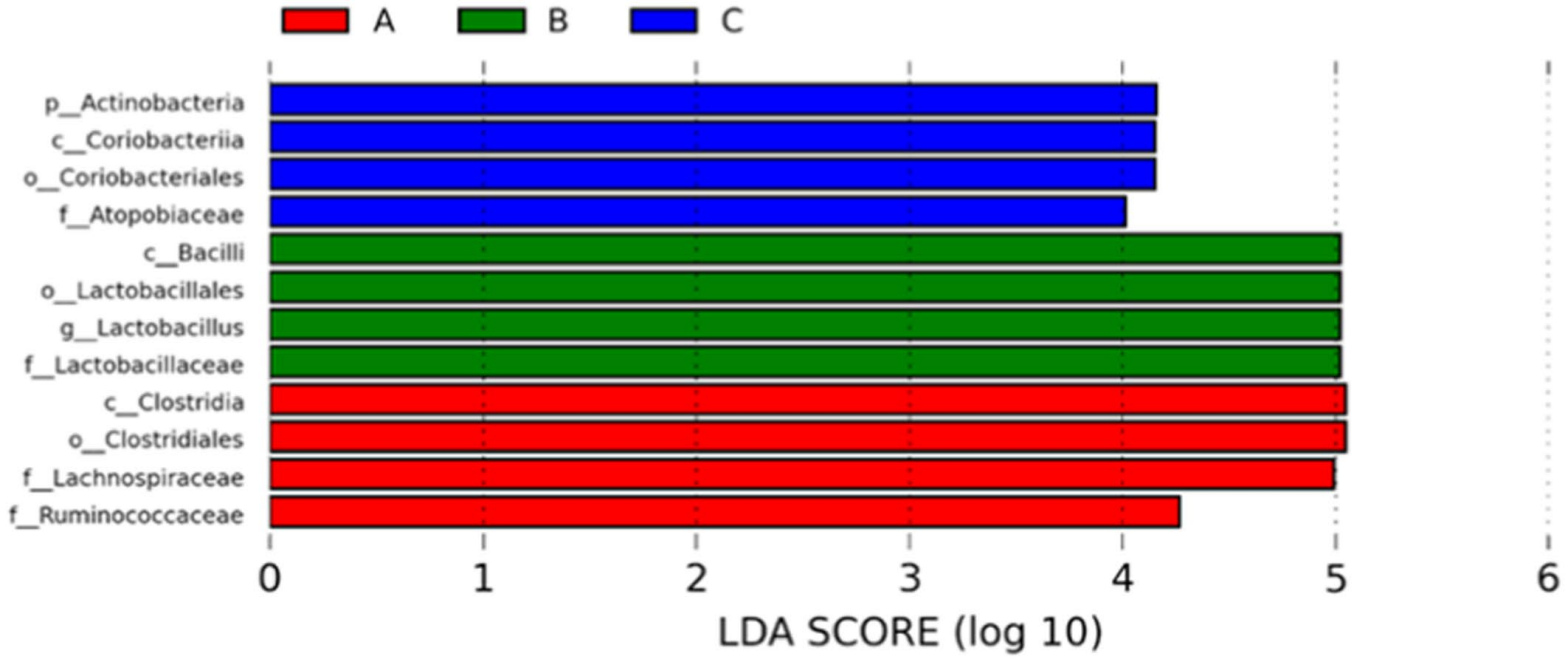
Use the non-parametric factorial Kruskal–Wallis (KW) sum-rank test to find species with significant differences in abundance by linear discriminant analysis (LDA) effect size (LEfSe) assay. Linear regression discriminant analysis (LDA) was used to estimate the magnitude of the effect of each species abundance on the difference. A: App*^NL-G-F/NL-G-F^* transgenic mice; B: App*^NL-G-F/NL-G-F^* transgenic mice administered with microcapsules (200 mg/kg bw) prepared from okra polysaccharides were mixed with sodium alginate (5%); C: App*^NL-G-F/NL-G-F^* transgenic mice administered with *L. plantarum*-encapsulated microcapsules (200 mg/kg bw) prepared from okra polysaccharides were mixed with sodium alginate (5%).

**Figure 8:**
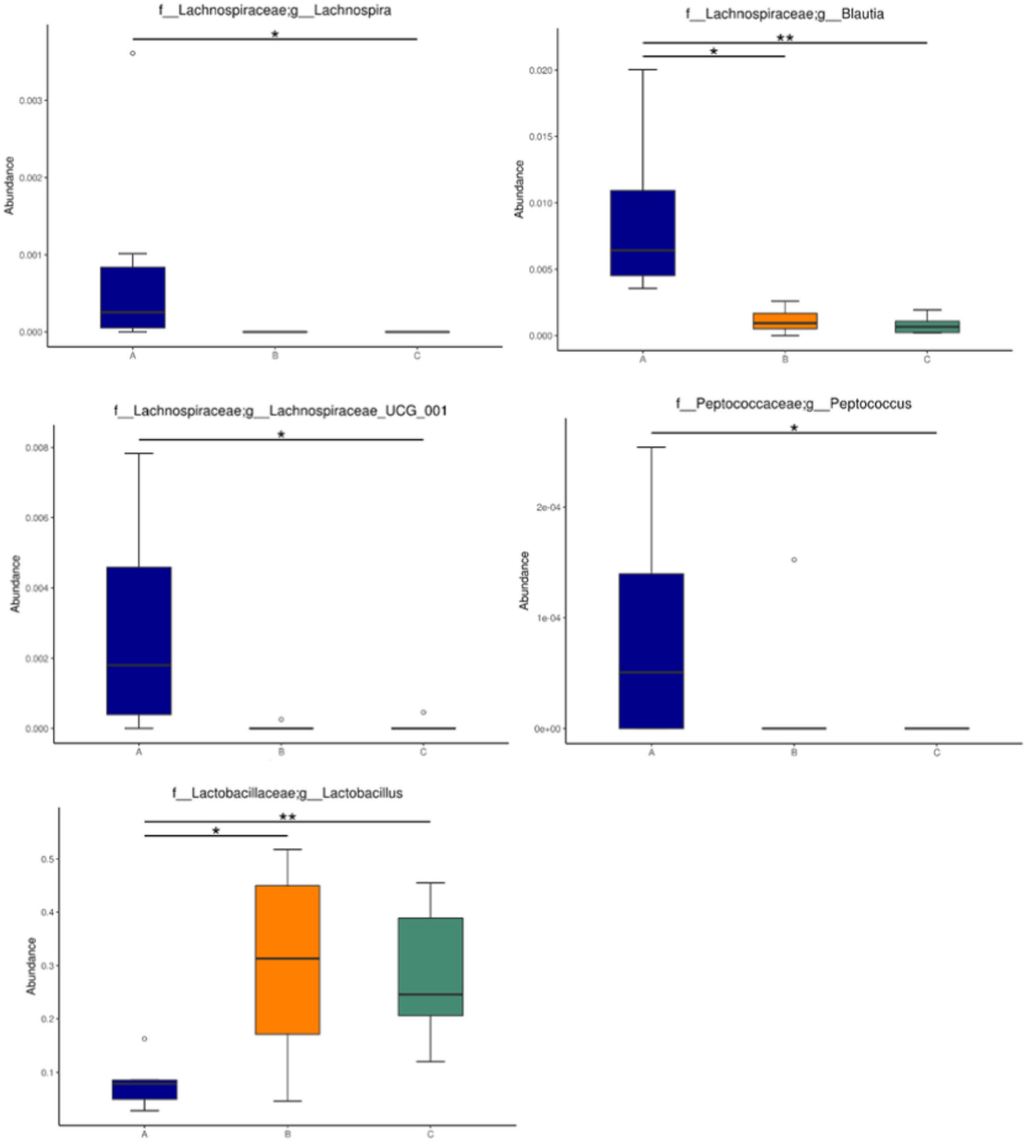
Relative abundance of the gut microbiota in mice administered with microcapsules prepared from okra polysaccharides with or without *L. plantarum* encapsulation. Abundance of *Lachnospira, Blautia, Lachnospiraceae*_UCG_001, *Peptococcus,* and *Lactobacillus* changed significantly among the groups. *p < 0.05 and **p < 0.01. A: App*^NL-G-F/NL-G-F^* transgenic mice; B: App*^NL-G-F/NL-G-F^* transgenic mice administered with microcapsules (200 mg/kg bw) prepared from okra polysaccharides were mixed with sodium alginate (5%); C: App*^NL-G-F/NL-G-F^* transgenic mice administered with *L. plantarum*-encapsulated microcapsules (200 mg/kg bw) prepared from okra polysaccharides were mixed with sodium alginate (5%).

The abundance of significant differences in species was evaluated by linear discriminant analysis (LDA) effect size (LEfSe) assay. The predominant microbial groups in AD mice including *Lachnospiraceae, Ruminococcaceae, Clostridia,* and *Clostridiales* are significantly elevated and potentially serve as biomarkers. However, these bacteria are all decreased in the gut of AD mice by administration with microcapsules prepared from okra polysaccharides with or without *L. plantarum* encapsulation, respectively (Figure 7). Furthermore, bacterial populations such as *Lachnospira, Blautia, Lachnospiraceae*_UCG_001, and *Peptococcus*, although not the most abundant in terms of richness, are notably suppressed by the administration with microcapsules prepared from okra polysaccharides with or without *L. plantarum* encapsulation (Figure 8). These results indicate that okra polysaccharides themselves possess the potential to regulate the gut microbiota of AD mice.

### 3.5 Effects of microencapsules prepared from okra polysaccharides with *Lactiplantibacillus plantarum* on fecal short-chain fatty acid (SCFA) in App*^NL-G-F/NL-G-F^* mice

We evaluated the fecal SCFA levels (including acetate, propionic acid, and butyrate) of AD mice after administering microcapsules prepared from okra polysaccharides with *L. plantarum* encapsulation (Table 2). Our results indicated that microcapsules prepared from okra polysaccharides with or without *L. plantarum* encapsulation markedly elevated acetate and propionic acid levels in AD mice, but only butyrate levels were increased by microcapsules prepared from okra polysaccharides with *L. plantarum* encapsulation. Similar to other studies, the SCFA levels *in vivo* were promoted by okra supplementation (Zhang et al., 2019).

**Table 2:** Productions of short-chain fatty acid productions in fecal of TG mice administered with okra polysaccharides or lactic acid bacteria-fermented okra polysaccharides.

Groups	Acetate	Propionic acid	Butyrate
	Concentration (μM)		
A	68.0 ± 6.1^c^	20.4 ± 0.8^b^	737.4 ± 103.6^b^
B	118.4 ± 8.4^b^	39.3 ± 4.5^a^	892.1 ± 81.0^b^
C	163.1 ± 13.8^a^	42.1 ± 3.7^a^	1224.7 ± 91.4^a^

Data are shown as mean ± SD (n = 3). Significant difference are indicated by different letters (a, b, and c).

## 4 Discussion

A bacterial amyloid known as curli is produced by *Escherichia coli*, and it is clearly seen through the sub-mock system that bacterial amyloids, such as prions, accelerate host Aβ aggregation (Zhou et al., 2012). Many other microorganisms have also been found to produce amyloids. For example, *Pseudomonas fluorescens* produces FapC, *Staphylococcus aureus* produces phenol-soluble modulins, *Streptomyces coelicolor* produces chaplins, and *Klebsiella pneumonia* produces MccE492, all of which are similar to human amyloid proteins (Schwartz and Boles, 2013). Studies have reported that extracellular Aβ produced by bacteria (*Escherichia coli*, *Salmonella enterica*, *Salmonella* Typhimurium, *Bacillus subtilis*, *Mycobacterium tuberculosis*, *Staphylococcus aureus*) also forms fibrous plaques after being released into the intestine (Pistollato et al., 2016). A study confirmed that amyloid proteins produced by microorganisms in the gut enter the central nervous system through the bloodstream and increase the accumulation and deposition of Aβ (Chen et al., 2016). A bacterial-derived amyloid called curli, which is produced by *Escherichia coli* and accelerates Aβ aggregation, similar to what occurs with prions, has been clearly observed in the sub-simulation system (Zhou et al., 2012). There are also many other microorganisms that have been found to produce amyloid-like peptides, for example, *Pseudomonas fluorescens* can produce FapC; *Staphylococcus aureus* can produce phenol-soluble modulins; *Streptomyces coelicolor* can produce chaplins; and *Klebsiella pneumonia* can produce MccE492. These proteins are considered to have human amyloid protein characteristics (Schwartz and Boles, 2013). Furthermore, other bacteria also generate extracellular Aβ, thereby forming fibrous plaques after being released, including *Escherichia coli*, *Salmonella enterica*, *Salmonella* Typhimurium, *Bacillus subtilis*, *Mycobacterium tuberculosis*, and *Staphylococcus aureus* (Pistollato et al., 2016).

Okra polysaccharides have been found to reverse metabolic disorders and cognitive function in AD mice and improve gut microbiota to protect against depression (Yan et al., 2020a,b). Many microorganisms in the gut affect host behavior and emotional performance by producing brain neurotransmitters (Foster, 2013). The probiotic bacteria *Bifidobacterium infantis*, when administered in germ-free mice by fecal transplantation or oral routes, can restore cranial nerve damage and cause behavioral changes (Sudo et al., 2004). In another experiment, the spatial memory and learning abilities of germ-free mice following fecal transplantation or probiotic treatment were significantly better compared with that of a control group (Gareau et al., 2011). Numerous studies have found that microbes in the gut may potentially interact to influence their growth or distribution (Zhang et al., 2013). These studies found that such changes in the intestinal microbiota could improve neuronal activity.

Microorganisms of *Lachnospiraceae* can cause diabetes in germ-free mice (Kameyama and Itoh, 2014). Therefore, microorganisms in this family may contain bacteria belonging to probiotics or opportunistic bacteria that cause disease. After feeding mice with lactic acid bacteria, the distribution of intestinal flora of the *Lachnospiraceae* NK4A136 group and *Lachnospiraceae* UCG-006 was increased and inhibited, respectively (Li et al., 2020), suggesting that the functions of various microorganisms within the *Lachnospiraceae* family may be opposing (Vacca et al., 2020). An increase in the gastric vagus nerve activity in association with *Lactobacillus johnsonii* has been reported (Tanida et al., 2005). Moreover, *Lactobacillus rhamnosus*-derived metabolites have the potential to ameliorate neuronal inflammation (Bravo et al., 2011). Recently, the effects of various polysaccharides on intestinal microbiota have been investigated, including polysaccharides derived from pitaya, *Cordyceps militaris*, and other edible fungal polysaccharides (Liang et al., 2021; Lee et al., 2021b, 2022; Sun et al., 2023).

Fecal microbiota transplantation or oral administration of the probiotic *Bifidobacterium infantis* to germ-free mice restored cranial nerve damage and led to behavioral changes (Sudo et al., 2004). In another experiment, germ-free mice that received fecal microbiota transplantation or probiotics had significantly better spatial memory and learning abilities than the controls (Gareau et al., 2011). A high bacterial phylum ratio of Firmicutes/Bacteroidetes can lead to intestinal inflammation and influence the attack rate of AD, whereas probiotics in the gut can reduce the bacterial growth of the Firmicutes phylum and increase the bacterial growth of the Bacteroidetes phylum, reducing the risk of AD (Gareau et al., 2011). Although researchers currently do not fully understand the role of intestinal flora in the prevention and control of AD in healthy people, it is also impossible to know whether AD symptoms lead to changes in intestinal flora, or whether intestinal flora themselves can influence the development of this disease. However, the above results show that intestinal bacteria play an extremely important role in the occurrence of AD by okra (Supplementary Figure S2). Taken together, probiotics in the intestinal flora can reduce the growth of *Firmicutes* and *Bacteroidetes* bacteria, consequently reducing the risk of AD (Cowan et al., 2014; Doroszkiewicz et al., 2021; Duan et al., 2021). In another study, a low-calorie diet promoted healthy gut flora, including bacteria of the genus *Lactobacillus* (Zhang et al., 2013).

The SCFAs were always produced from *Lachnospiraceae* (Vacca e al., 2020) and *Ruminococcaceae* (Xie et al., 2022), but the levels of these two bacteria were lowered in TG mice treated with samples. We found that the *Lactobacillaceae* was elevated by okra polysaccharide microcapsules with or without encapsulated-lactic acid bacteria. Recently, lactic acid bacteria that generated SCFAs *in vitro* (Kang et al., 2021) and promoted intestinal SCFAs *in vivo* (Ni et al., 2021) have been reported. We hypothesized that okra polysaccharides may be as prebiotics for upregulating lactic acid bacteria growth and SCFA production.

Taken together, the results showed that the microcapsules prepared from okra polysaccharides with or without *L. plantarum* encapsulation had similar effects on regulating *Lactobacillus* population in AD mice. Moreover, the microcapsules prepared from okra polysaccharides with or without *L. plantarum* encapsulation also elevated intestinal SCFA level of AD mice. According to the above, microcapsules prepared from okra polysaccharides with or without *L. plantarum* encapsulation have the potential to act as a dietary supplement and health food via intestinal regulation.

## Data availability statement

The raw data supporting the conclusions of this article will be made available by the authors, without undue reservation.

## Ethics statement

The animal study was approved by National Cheng Kung University in Taiwan with IACUC approval no. 108317. The study was conducted in accordance with the local legislation and institutional requirements.

## Author contributions

Y-KH: Data curation, Formal analysis, Funding acquisition, Writing – original draft. B-HL: Writing – original draft, Writing – review & editing. S-CW: Funding acquisition, Investigation, Methodology, Project administration, Writing – review & editing.

## Footnotes

^1^v2019.7.0; https://qiime2.org/

## References

[ref1] BeMiller J. N. Whistler R. L. Barbalowm D. G. Chen C. C. (1993). “Aloe, Chia, Flaxseed, Okra, psyllium seed, quince seed, and tamarind gums” in Industrial gums. Polysaccharide and their derivatives. eds. Roy L. W. BeMiller J. N.. 3rd ed (San Diago: Academic Press), 235–255.

[ref2] Bravo J. A. Forsythe P. Chew M. V. Escaravage E. Savignac H. M. Dinan T. G.. (2011). Ingestion of Lactobacillus strain regulates emotional behavior and central GABA receptor expression in a mouse via the vagus nerve. Proc. Natl. Acad. Sci. U. S. A. 108, 16050–16055. doi: 10.1073/pnas.1102999108, PMID: 21876150 PMC3179073

[ref3] Chen S. G. Stribinskis V. Rane M. J. Demuth D. R. Gozal E. Roberts A. M.. (2016). Exposure to the functional bacterial amyloid protein curli enhances alpha-synuclein aggregation in aged Fischer rats and *Caenorhabditis elegans*. Sci. Rep. 6:34477. doi: 10.1038/srep34477, PMID: 27708338 PMC5052651

[ref4] Cowan T. E. Palmnas M. S. A. Yang J. Bomhof M. R. Ardell K. A. Reimer R. A.. (2014). Chronic coffee consumption in the diet-induced obese rat: impact on gut microbiota and serum metabolomics. J. Nutr. Biochem. 25, 489–495. doi: 10.1016/j.jnutbio.2013.12.009, PMID: 24629912

[ref5] De la Cuesta-Zuluaga J. Mueller N. T. Corrales-Agudelo V. Escobar J. S. (2017). Metformin is associated with higher relative abundance of mucin-degrading *Akkermansia muciniphila* and several short-chain fatty acid producing microbiota in the gut. Diabetic Care 40, 54–62. doi: 10.2337/dc16-1324, PMID: 27999002

[ref6] Doroszkiewicz J. Groblewska M. Mroczko B. (2021). The role of gut microbiota and gut-brain interplay in selected diseases central nervous system. Int. J. Mol. Sci. 22:10028. doi: 10.3390/ijms221810028, PMID: 34576191 PMC8471822

[ref7] Duan M. Liu F. Fu H. Lu S. Wang T. (2021). Preoperative microbiomes and intestinal barrier function can differentiate prodromal Alzheimer's disease from normal neurocognition in elderly patients scheduled to undergo orthopedic surgery. Front. Cell. Infect. Microbiol. 11:592842. doi: 10.3389/fcimb.2021.592842, PMID: 33869072 PMC8044800

[ref8] Dubois M. Gilles K. A. Hamilton J. K. Rebers P. T. Smith F. (1956). Colorimetric method for determination of sugars and related substances. Anal. Chem. 28, 350–356. doi: 10.1021/ac60111a017

[ref9] Foster J. A. (2013). Gut feelings: bacteria and the brain. Cerebrum 2013:9. PMID: 24116266 PMC3788166

[ref10] Gareau M. G. Wine E. Rodrigues D. M. Cho J. H. Whary M. T. Philpott D. J.. (2011). Bacterial infection causes stress-induced memory dysfunction in mice. Gut 60, 307–317. doi: 10.1136/gut.2009.202515, PMID: 20966022

[ref11] Huang C. N. Lin C. L. Li H. H. Tsou S. H. Peng C. H. (2023). *Abelmoschus esculentus* (okra) prevents insulin resistance and restores neuron autophagy by regulating dipeptidyl peptidase-4 and thus improving hippocampal function. J. Med. Food 26, 462–469. doi: 10.1089/jmf.2023.K.0014, PMID: 37358589

[ref12] Huang C. N. Wang C. J. Lin C. L. Li H. H. Yen A. T. Peng C. H. (2020). *Abelmoschus esculentus* subfractions attenuate Aβ and tau by regulating DPP-4 and insulin resistance signals. BMC Complement. Med. Ther. 20:370. doi: 10.1186/s12906-020-03163-4, PMID: 33267804 PMC7709418

[ref13] Kameyama K. Itoh K. (2014). Intestinal colonization by a Lachnospiraceae bacterium contributes to the development of diabetes in obese mice. Microb. Environ. 29, 427–430. doi: 10.1264/jsme2.ME14054, PMID: 25283478 PMC4262368

[ref14] Kang C. J. Kim K. S. Park H. M. Kim S. Paek N. S. (2021). Antioxidant activity and short-chain fatty acid production of lactic acid bacteria isolated from Korean individuals and fermented foods. 3 Biotech 11:217. doi: 10.1007/s13205-021-02767-y PMC8050147 33936926

[ref15] Kuo H. C. Kwong H. K. Chen H. Y. Hsu H. Y. Yu S. H. Hsieh C. W.. (2021). Enhanced antioxidant activity of Chenopodium formosanum Koidz. By lactic acid bacteria: optimization of fermentation conditions. PLoS One 16:e0249250. doi: 10.1371/journal.pone.0249250, PMID: 33974647 PMC8112705

[ref16] Lee B. H. Chen C. H. Hsu Y. Y. Chuang P. T. Shih M. K. Hsu W. H. (2021a). Polysaccharides obtained from *Cordyceps militaris* alleviate hyperglycemia by regulating gut microbiota in mice fed a high-fat/sucrose diet. Food Secur. 10:1870. doi: 10.3390/foods10081870 PMC8391476 34441649

[ref17] Lee B. H. Hsu K. T. Chen Y. Z. Tain Y. L. Hou C. Y. Lin Y. C.. (2022). Polysaccharide extracts derived from defloration waste of fruit pitaya regulates gut microbiota in a mice model. Fermentation 8:108. doi: 10.3390/fermentation8030108

[ref18] Lee B. H. Hsu W. H. Chien H. Y. Hou C. Y. Hsu Y. T. Chen Y. Z.. (2021b). Applications of *Lactobacillus acidophilus*-fermented mango protected *Clostridioides difficile* infection and developed as an innovative probiotic jam. Food Secur. 10:1631. doi: 10.3390/foods10071631, PMID: 34359501 PMC8303244

[ref19] Lee B. H. Huang S. C. Hou C. Y. Chen Y. Z. Chen Y. H. Hazeena S. H.. (2023). Effect of polysaccharide derived from dehulled adlay on regulating gut microbiota and inhibiting *Clostridioides diffcile* in an vitro colonic fermentation model. Food Chem. 410:135410. doi: 10.1016/j.foodchem.2023.135410, PMID: 36628918

[ref20] Lengsfeld C. Titgemeyer F. Faller G. Hensel A. (2004). Glycosylated compounds from okra inhibit adhesion of *Helicobacter pylori* to human gastric mucosa. J. Agric. Food Chem. 52, 1495–1503. doi: 10.1021/jf030666n, PMID: 15030201

[ref21] Li H. Liu F. Lu J. Shi J. Gua J. Yan F.. (2020). Probiotic mixture of *Lactobacillus plantarum* strains improves lipid metabolism and gut microbiota structure in high fat diet fed mice. Front. Microbiol. 11:512. doi: 10.3389/fmicb.2020.00512, PMID: 32273874 PMC7113563

[ref22] Liang J. Zhang M. Wang X. Ren Y. Yue T. Wang Z.. (2021). Edible fungal polysaccharides, the gut microbiota, and host health. Carbohydr. Polym. 273:118558. doi: 10.1016/j.carbpol.2021.118558, PMID: 34560969

[ref23] Liao Z. Zhang J. Liu B. Yan T. Xu F. Xiao F.. (2019). Polysaccharide from okra [*Abelmoschus esculentus* (L.) Moench] improves antioxidant capacity via PI3K/Akt pathways and Nrf2 translocation in a type 2 diabetes model. Molecules 24:1906. doi: 10.3390/molecules24101906 31108940 PMC6571734

[ref24] Lin C. Hung W. T. Kuo C. Y. Liao K. S. Liu Y. C. Yang W. B. (2010). I2-catalyzed oxidative condensation of aldoses with diamines: synthesis of aldonaphthimidazoles for carbohydrate analysis. Molecules 15, 1340–1353. doi: 10.3390/molecules15031340, PMID: 20335985 PMC6257232

[ref25] Liu C. F. Pan T. M. (2010). In vitro effects of lactic acid bacteria on cancer cell viability and antioxidant activity. J. Food Drug Anal. 18, 77–86.

[ref26] Machiels K. Joossens M. Claes K. Verhaegen J. Rutgeerts P. Vermeire S. (2014). A decrease of the butyrate-producing species *Roseburia hominis* and *Faecalibacterium prausnitzii* defines dysbiosis in patients with ulceratie colitis. Gut 63, 1275–1283. doi: 10.1136/gutjnl-2013-304833, PMID: 24021287

[ref27] Nasiri H. Golestan L. Shahidi S. A. Darjani P. (2021). Encapsulation of *Lactobacillus casei* in sodium alginate microcapsules: improvement of the bacterial viability under simulated gastrointestinal conditions using wild sage seed mucilage. J. Food Meas. Charact. 15, 4726–4734. doi: 10.1007/s11694-021-01022-5

[ref28] Ni C. Li X. Wang L. Li X. Zhao J. Zhang H.. (2021). Lactic acid bacteria strains relieve hyperuricaemia by suppressing xanthine oxidase activity via a short-chain fatty acid-dependent mechanism. Food Funct. 12, 7054–7067. doi: 10.1039/D1FO00198A 34152353

[ref29] Pistollato F. Sumalla Cano S. Elio I. Masias Vergara M. Giampieri F. Battino M. (2016). Role of gut microbiota and nutrients in amyloid formation and pathogenesis of Alzheimer disease. Nutr. Rev. 74, 624–634. doi: 10.1093/nutrit/nuw023 27634977

[ref30] Qin J. Li R. Raes J. Arumugam M. Burgdorf K. S. Manichanh C.. (2020). A human gut microbial gene catalogue established by metagenomic sequencing. Nature 464, 59–65. doi: 10.1038/nature08821 PMC3779803 20203603

[ref31] Rajilic-Stojanovic M. Biagi E. Heilig H. G. Kajander K. Kellonen R. A. Tims S.. (2011). Global and deep molecular analysis of microbiota signatures in fecal samples from patients with irritable bowel syndrome. Gastroenterology 141, 1792–1801. doi: 10.1053/j.gastro.2011.07.043, PMID: 21820992

[ref32] Ralet M. C. Della G. J. F. (1993). Thibault: raw and extruded fibre from pea hulls. Part I: composition and physico-chemical properties. Carbohyd. Polym. 20, 17–23. doi: 10.1016/0144-8617(93)90028-3

[ref33] Romanchik-Cerpovicz J. E. Tilmon R. W. Baldree K. A. (2002). Moisture retention and consumer acceptability of chocolate bar cookies prepared with okra gum as a fat ingredient substitute. J. Am. Diet. Assoc. 102, 1301–1303. doi: 10.1016/S0002-8223(02)90287-7, PMID: 12792632

[ref34] Sakakibara Y. Sekiya M. Saito T. Saido T. C. Iijima K. M. (2018). Cognitive and emotional alterations in app knock-in mouse models of Aβ amyloidosis. BMC Neurosci. 19:46. doi: 10.1186/s12868-018-0446-8, PMID: 30055565 PMC6064053

[ref35] Sami R. Lianzhou J. Yang L. Ma Y. Jing J. (2013). Evaluation of fatty acid and amino acid compositions in okra (*Abelmoschus esculentus*) grown in different geographical locations. Biomed. Res. Int. 2013:574283, 1–6. doi: 10.1155/2013/574283 24171167 PMC3793589

[ref36] Schwartz K. Boles B. R. (2013). Microbial amyloids–functions and interactions within the host. Curr. Opin. Microbiol. 16, 93–99. doi: 10.1016/j.mib.2012.12.001, PMID: 23313395 PMC3622111

[ref37] Sengkhamparn N. Verhoef R. Schols H. A. Sajjaanantakul T. Voragen A. G. (2009). Characteration of cell wall polysaccharides from okra [*Abelmoschus esculentus* (L.) Moench]. Carbohydr. Res. 344, 1824–1832. doi: 10.1016/j.carres.2008.10.012, PMID: 19061990

[ref38] Soscia S. J. Kirby J. E. Washicosky K. J. Tucker S. M. Ingelsson M. Hyman B.. (2010). The Alzheimer’s disease-associated amyloid beta-protein is an antimicrobial peptide. PLoS One 5:e9505. doi: 10.1371/journal.pone.0009505, PMID: 20209079 PMC2831066

[ref39] Sudo N. Chida Y. Aiba Y. Sonoda J. Oyama N. Yu X. N.. (2004). Postnatal microbial colonization programs the hypothalamic-pituitary-adrenal system for stress response in mice. J. Physiol. 558, 263–275. doi: 10.1113/jphysiol.2004.063388, PMID: 15133062 PMC1664925

[ref40] Sun Y. Ho C. T. Zhang Y. Hong M. Zhang X. (2023). Plant polysaccharides utilized by gut microbiota: new players in ameliorating cognitive impairment. J. Tradit. Complement. Med. 13, 128–134. doi: 10.1016/j.jtcme.2022.01.003, PMID: 36970456 PMC10037067

[ref41] Tanida M. Yamano T. Maeda K. Okumura N. Fukushima Y. Nagai K. (2005). Effects of intraduodenal injection of *Lactobacillus johnsonii* La1 on renal sympathetic nerve activity and blood pressure in urethane-anesthetized rats. Neurosci. Lett. 389, 109–114. doi: 10.1016/j.neulet.2005.07.036, PMID: 16118039

[ref42] Turner J. R. (2009). Intestinal mucosal barrier function in health and disease. Nat. Rev. Immunol. 9, 799–809. doi: 10.1038/nri2653 19855405

[ref43] Vacca M. Celano G. Calabrese F. M. Portincasa P. Gobbett M. de Angelis M. (2020). The controversial role of human gut Lachnospiraceae. Microorganisms 8:573. doi: 10.3390/microorganisms8040573, PMID: 32326636 PMC7232163

[ref44] Whistler R. L. Conrad H. E. (1954). 2-O-(D-galactopyranosyluronic acid)-L-rhamnose from okra mucilage. J. Am. Chem. Soc. 76, 3544–3546. doi: 10.1021/ja01642a057

[ref45] Woolfe M. L. Chaplin M. F. Otchere G. (1977). Studies on the mucilages extracted from okra fruit (*Hibiscus eaculentus* L.) and baobab (*Adansonia* L.). J. Sci. Food Agric. 28, 519–529. doi: 10.1002/jsfa.2740280609

[ref46] Xie J. Li L. F. Dai T. Y. Qi X. Wang Y. Zheng T. Z.. (2022). Short-chain fatty acids produced by Ruminococcaceae mediate α-linolenic acid promote intestinal stem cells proliferation. Mol. Nutr. Food Res. 66:e2100408. doi: 10.1002/mnfr.202100408 34708542

[ref47] Yan T. Nian T. Liao Z. Xiao F. Wu B. Bi K.. (2020a). Antidepressant effects of a polysaccharide form okra [*Abelmoschus esculentus* (L) Moench] by anti-inflammation and rebalancing the gut microbiota. Int. J. Biol. Macromol. 144, 427–440. doi: 10.1016/j.ijbiomac.2019.12.138, PMID: 31862370

[ref48] Yan T. Nian T. Wu B. Xiao F. He B. Bi K.. (2020b). Okra polysaccharides can reverse the metabolic disorder induced by high-fat diet and cognitive function injury in Aβ1-42 mice. Exp. Gerontol. 130:110802. doi: 10.1016/j.exger.2019.110802 31794852

[ref49] Zhang C. Li S. Yang L. Huang P. Li W. Wang S.. (2013). Structural modulation of gut microbiota in life-long calorie-restricted mice. Nat. Commun. 4:2163. doi: 10.1038/ncomms3163, PMID: 23860099 PMC3717500

[ref50] Zhang J. Zhao Y. Ren D. Yang X. (2019). Effect of okra fruit powder supplementation on metabolic syndrome and gut microbiota diversity in high fat diet-induced obese mice. Food Res. Int. 130:108929. doi: 10.1016/j.foodres.2019.108929 32156377

[ref51] Zhou Y. Smith D. Leong B. J. Brännström K. Almqvist F. Chapman M. R. (2012). Promiscuous cross-seeding between bacterial amyloids promotes interspecies biofilms. J. Biol. Chem. 287, 35092–35103. doi: 10.1074/jbc.M112.383737, PMID: 22891247 PMC3471717

