# Supplementary material for: Lactiplantibacillus plantarum-encapsulated microcapsules prepared from okra polysaccharides improved intestinal microbiota in Alzheimer’s disease mice
Source: Front Microbiol. 2024 Mar 18;15:1305617. doi: 10.3389/fmicb.2024.1305617 (PMC10982412; doi:10.3389/fmicb.2024.1305617)
Supplement: Supplementary file 1 [file Data_Sheet_1.docx]

Supplementary Material

**Supplementary Table 1.** Effect of different sodium alginate concentrations on moisture content and particle size of okra polysaccharides microcapsules


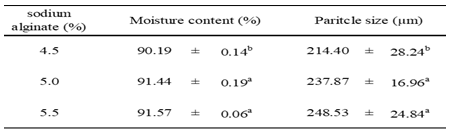


*Each of moisture content is expressed as mean ± S.D. (n=3).

* Particle size is expressed as mean ± S.D. (n=15).

Each value on the top right of means in the same column bearing of different letters is significantly different (*p*<0.05).


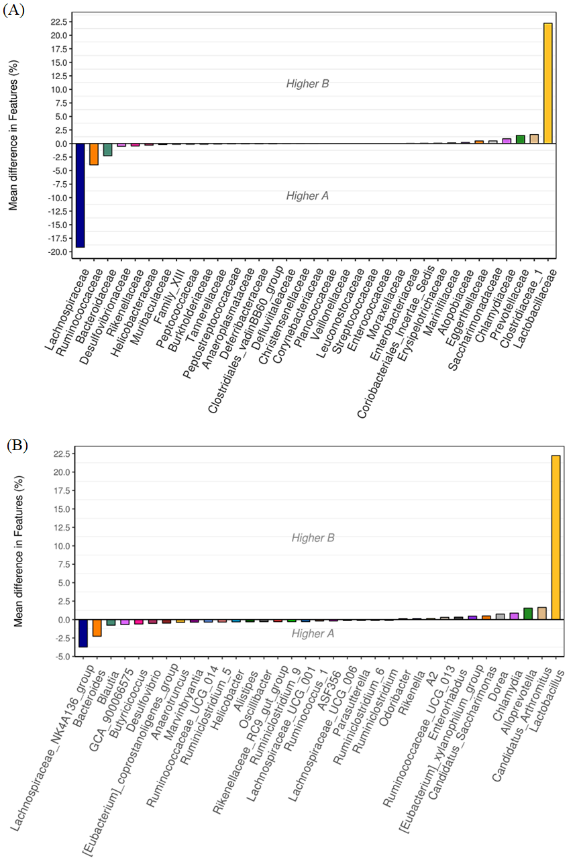


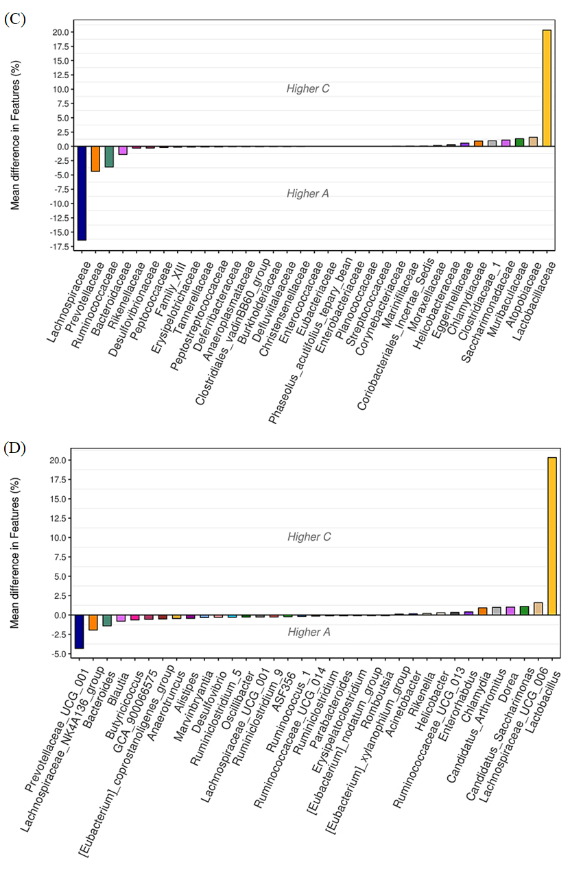


**Supplementary Figure 1.** Mean difference in features at (A) Family and (B) Genus between group A and group B. Mean difference in features at (C) Family and (D) Genus between group A and group C.

A: APP*^NL-G-F/NL-G-F^* transgenic mice

B: APP*^NL-G-F/NL-G-F^* transgenic mice administered with microcapsules (200 mg/kg bw) preparated by okra polysaccharides were mixed with sodium alginate (5%)

C: APP*^NL-G-F/NL-G-F^* transgenic mice administered with *L. plantarum*-encapsulated microcapsules (200 mg/kg bw) preparated by okra polysaccharides were mixed with sodium alginate (5%).

**
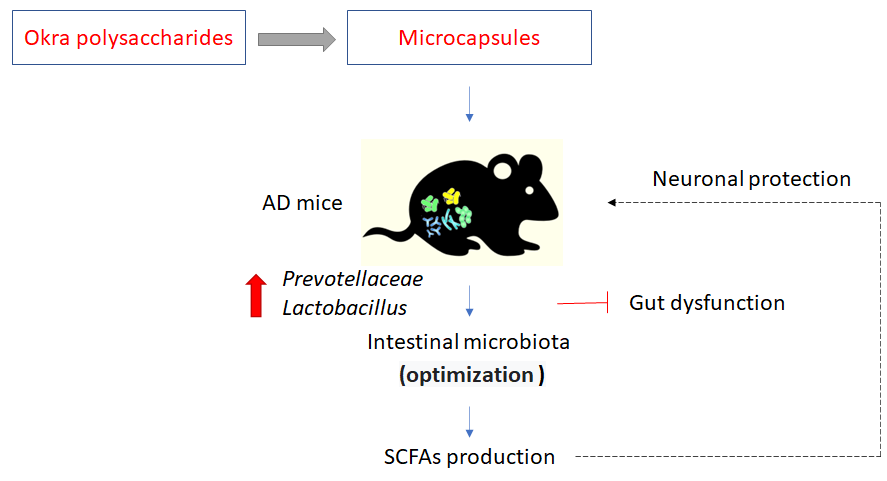
**

**Supplementary Figure 2.** The graphical abstract for neuronal protection of administered with okra polysaccharides preparated-microcapsules with or without *L. plantarum* encapsulation.

**
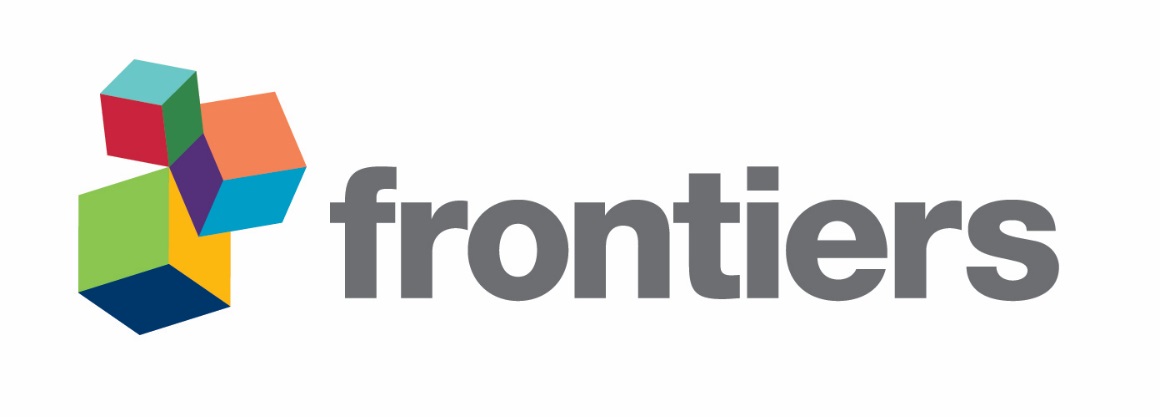
**

**Supplementary Figure 1.** The figure legends are required to have the same font as the main text, 12 point normal Times New Roman, single spaced. Please use a single paragraph for each legend and prepare the figures keeping in mind the PDF layout.
